# Supplementary material for: Molecular Infectious Disease Epidemiology: Survival Analysis and Algorithms Linking Phylogenies to Transmission Trees
Source: PLoS Comput Biol. 2016 Apr 12;12(4):e1004869. doi: 10.1371/journal.pcbi.1004869 (PMC4829193; doi:10.1371/journal.pcbi.1004869)
Supplement: S1 Appendix — (PDF) [file pcbi.1004869.s001.pdf]

Appendix

Molecular Infectious Disease Epidemiology: Survival Analysis and Algorithms Linking Algorithms Linking Phylogenies to Transmission Trees

Eben Kenah, Tom Britton, M. Elizabeth Halloran,
and Ira M. Longini, Jr.

PLoS Computational Biology 2016

Assumptions As stated in the main text, our assumptions are:

1. Each individual is infected at most once.

2. Each infection is initiated by a single pathogen. Following infection, within-host pathogen evolution occurs and the evolved pathogens are transmitted to others.

3. The order in which infections (or onsets of infectiousness) occurred is known.

4. We have at least one pathogen sequence from each infected individual, and these sequences are linked in a rooted phylogeny. The root of this phylogeny has a parent node r_0.

5. Each node in the phylogeny represents a pathogen that had a host, which is also the "host" of the node. A parent-child relationship between nodes with different hosts represents a direct transmission of infection from the host of the parent to the host of the child. The node r_0 has a host outside the observed population.

Lemma 1. *The nodes hosted by an infected individual form a subtree of the phylogenetic tree.*

Proof. This is trivial if i hosts only one node, so assume i hosts distinct nodes z and z'. Since the phylogeny is a rooted tree, there is a unique path from z to the root node r_0. Let x be the first node on this path such that $\text{host}(x) \neq i$. Similarly, let x' be the first node on the path from z' to r_0 such that $\text{host}(x') \neq i$. If $x' \neq x$, there are two possibilities:

1. If $\text{host}(x') \neq \text{host}(x)$, then i was infected by two different individuals, violating Assumption 1.

2. If $\text{host}(x') = \text{host}(x)$, then i was infected with two distinct pathogens, violating Assumption 2.

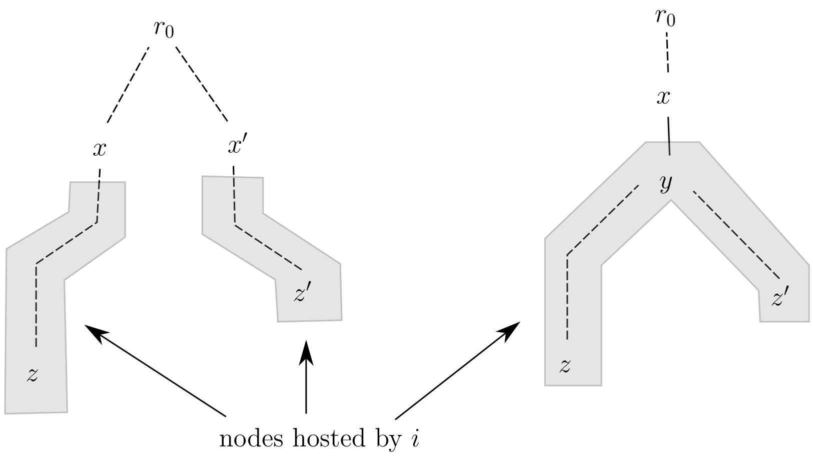

Figure 1: Illustration for Lemma 1 and Theorem 1. The left shows the paths from z and z' to x and x', respectively. On the right is the situation after we prove that $x' = x$. Solid lines indicate direct parent-child relationships, and dotted lines indicate paths of length ≥ 0. The infector of i must be host(x).

Therefore, $x' = x$. By definition, x has at least one child y such that $\text{host}(y) = i$. Since host(x) infected i exactly once, there can be at most one such child of x. If z is any node hosted by i, there is a path from z to y that consists entirely of nodes hosted by i. Therefore, the nodes hosted by i form a phylogenetic subtree rooted at y. See Figure 1. □

Theorem 1. *A phylogeny with known interior node hosts implies a unique transmission tree.*

Proof. Choose an infected individual i. By Lemma 1, the nodes hosted by i form a subtree of the phylogeny. Let y be the root of this subtree and let $x = \text{parent}(y)$. Since $\text{host}(x) \neq i$, host(x) infected i by Assumption 5. Therefore, the infector of each i is uniquely determined by the phylogeny and the interior node hosts. □

Lemma 2. *For any node x, host(x) = first(x) or host(x) infected first(x).*

Proof. Let $j = \text{first}(x)$, and let r_j be the root of the subtree consisting of nodes hosted by j. There are three cases:

1. If $r_j = x$, then $\text{host}(x) = \text{host}(r_j) = j$.
2. If $r_j \notin C_x$, let ℓ_j be a leaf in C_x hosted by j. The phylogeny is a tree and x is the root of the clade C_x, so any path from a node outside C_x to a node in C_x must include x. Since all nodes on the path from r_j to ℓ_j are hosted by j, $\text{host}(x) = j$. See the left side of Figure 2.

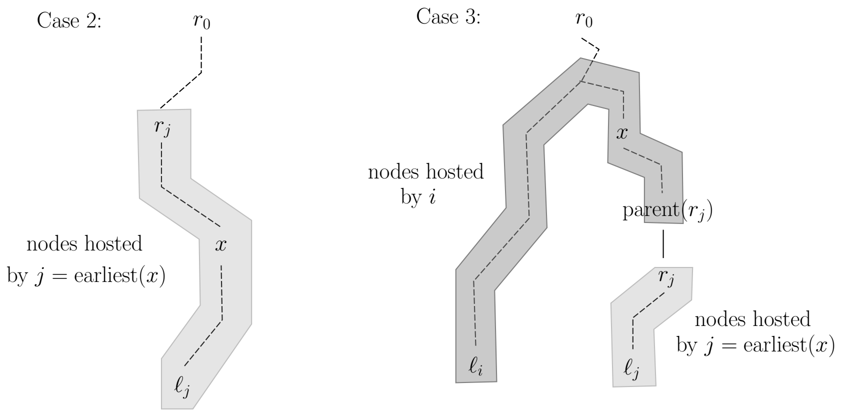

Figure 2: Illustrations for Lemma 2. The left shows Case 2, where r_j is outside C_x and $\text{host}(x) = j$. The right shows Case 3, where r_j is inside C_x and $\text{host}(x) = i$, where i is the infector of j. As in Figure 1, solid lines indicate parent-child relationships and dotted lines indicate paths of length ≥ 0.

3. If $r_j \in C_x$ and $r_j \neq x$, let $i = \text{host}\big(\text{parent}(r_j)\big)$ be the infector of j.

   (a) If $i = 0$, then $i = \text{host}(r_0)$.

   (b) If $i \neq 0$, then i is the host of a leaf ℓ_i. Since i was infected before j, ℓ_i must be outside C_x.

   Either way, there is a path from node y outside C_x to $\text{parent}(r_j)$ that consists of nodes hosted by i. Since $\text{parent}(r_j) \in C_x$, this path includes x so $\text{host}(x) = i$. See the right side of Figure 2.

Therefore, $\text{host}(x) = j$ or $\text{host}(x) = v_j$, where $j = \text{first}(x)$. □

Theorem 2. *A transmission tree corresponds to at most one possible assignment of interior node hosts in a phylogeny.*

Proof. Choose an interior node x of the phylogeny Φ and assume the transmission tree $\mathbf{v}$ is known. If $\text{first}(y) = \text{first}(x)$ for all children y of x, then each clade rooted at a child of x contains a leaf hosted by x, so $\text{host}(x) = \text{first}(x)$ by Lemma 1. Now suppose there is a child y of x such that $\text{first}(y) \neq \text{first}(x)$. By Lemma 2 applied to node x, $\text{host}(x) = v_{\text{first}(y)}$. Thus, $\text{host}(x)$ is uniquely determined by $\text{first}(y)$ and $v_{\text{first}(y)}$ for all children y of x. Since $\text{first}(y)$ is determined by Φ and $v_{\text{first}(y)}$ is determined by $\mathbf{v}$, there is at most one assignment of interior node hosts in Φ that will produce $\mathbf{v}$. □

Lemma 3. *If x is an interior node, $\text{host}(x) = \text{first}(x)$ or $\text{host}(x) = \text{host}\big(\text{parent}(x)\big)$.*

Proof. Suppose $\text{host}(y) \neq \text{first}(y)$. Then $\text{host}(y)$ infected $\text{first}(y)$ by Lemma 2. Since the nodes hosted by $\text{host}(y)$ form a subtree by Lemma 1 and $\text{host}(y)$ is the host of a leaf outside C_y, we must have $\text{host}(x) = \text{host}(y)$. □

Lemma 4. *If x is an interior node with child y in the phylogeny, then*

$$host(x) \in D_y^* = \begin{cases} D_y & \text{if } first(y) \notin D_y, \\ D_y \cup \mathcal{V}_{first(y)} & \text{if } first(y) \in D_y. \end{cases} \tag{1}$$

Proof. By Lemma 3, either $\text{host}(y) = \text{first}(y)$ or $\text{host}(y) = \text{host}(x)$. We consider two cases:

1. If $\text{first}(y) \notin D_y$, then $\text{host}(y) = \text{host}(x)$ so $\text{host}(x) \in D_y$.
2. If $\text{first}(y) \in D_y$, suppose $\text{host}(x) \notin D_y$. By Lemma 3, $\text{host}(y) = \text{first}(y)$. By Assumption 5, $\text{host}(x)$ infected $\text{first}(y)$. Thus, $\text{host}(x) \in D_y \cup \mathcal{V}_{\text{first}(y)}$.

Therefore, $\text{host}(x) \in D_y^*$ as defined in equation (1). □

Theorem 3. *For any interior node x in the phylogeny,*

$$D_x = \bigcap_{y \in children(x)} D_y^*, \tag{2}$$

where $children(x)$ denotes the children of x.

Proof. Since Lemma 4 holds for each child of x, we have $D_x \subseteq \bigcap_y D_y^*$. Now suppose $h \in \bigcap_y D_y^*$. When $h \in D_y$, there is at least one possible transmission tree within clade C_y that can be generated with $\text{host}(y) = h$. When $h \notin D_y$, then we must have $h \in \mathcal{V}_{\text{first}(y)}$ and $\text{first}(y) \in D_y$. For each child y of x, set

$$\text{host}(y) = \begin{cases} h & \text{if } h \in D_y, \\ \text{first}(y) & \text{if } h \notin D_y. \end{cases} \tag{3}$$

Using this choice of $\text{host}(y)$, we can generate a possible transmission tree within clade C_y for each child y of x. If $\text{host}(y) = h$, this transmission tree is rooted at h. Otherwise, it is rooted at $\text{first}(y)$ and we can add an edge from h to $\text{first}(y)$ because $h \in \mathcal{V}_{\text{first}(y)}$. These transmission trees rooted at h can be combined into a transmission tree within C_x that can be generated with $\text{host}(x) = h$. Thus $\bigcap_y D_y^* \subseteq D_x$, so the sets must be equal. □

Theorem 4. $H_x = A_x \cap D_x$.

Proof. Since D_x contains all nodes that satisfy the descendant constraints, $H_x \subseteq D_x$. By Lemma 3, $H_x \subseteq A_x$. Therefore, $H_x \subseteq A_x \cap D_x$. Now choose $h \in A_x \cap D_x$. Since $h \in D_x$, there is at least one possible transmission tree $\mathbf{v}_x$ within C_x that is rooted at h and has an edge ending in each member of $L_x \setminus \{h\}$. Since $h \in A_x$, there are two cases:

1. If $h \in H_{\text{parent}(x)}$, there is at least one possible transmission tree $\mathbf{v}_0$ produced when $\text{host}\big(\text{parent}(x)\big) = h$.

2. If $h \notin H_{\text{parent}(x)}$, then $h = \text{first}(x)$. Let $g = \text{host}\big(\text{parent}(x)\big)$. By Lemma 3, $\text{host}(x) = g$ or $\text{host}(x) = h$. If $\text{host}(x) = g$, then g infected h by Lemma 2. If $\text{host}(x) = h$, then g infected h by Assumption 5. Therefore, $g \in \mathcal{V}_h$. Since $g \in H_{\text{parent}(x)}$, we can set $\text{host}\big(\text{parent}(x)\big) = g$ and generate possible transmission tree $\mathbf{v}_0$ that has an edge from g to h.

For each $i \in L_x \setminus \{h\}$, replace its incoming edge in $\mathbf{v}_0$ with its incoming edge in $\mathbf{v}_x$. This generates a possible transmission tree $\mathbf{v}_1$ that can be generated when $\text{host}(x) = h$, so $h \in H_x$. Thus $A_x \cap D_x \subseteq H_x$, so the sets must be equal. □

Input: Rooted phylogeny Φ and epidemiologic data
Output: H_x for each node x of Φ
for *node x in postorder traversal of* Φ **do**
  if *x is a leaf* **then** $D_x = \{\text{host}(x)\}$;
  else $D_x = \cap_{y \in \text{children}(x)} D_y^*$, where D_y^* is defined in equation (1);
end
for *node x in preorder traversal of* Φ **do**
  if $x = r_0$ **then** $H_x = \{0\}$;
  else $H_x = D_x \cap A_x$, where $A_x = H_{\text{parent}(x)} \cup \{\text{first}(x)\}$;
end

Algorithm 1: Finding host sets.

Input: Rooted phylogeny Φ with nonempty H_x for each node x
Output: Transmission tree $\mathbf{v}$ simultaneously consistent with Φ and epidemiologic data
for *node x in preorder traversal of* Φ **do**
  if $x = r_0$ **then** set $\text{host}(x) = 0$;
  else
    $w = \text{parent}(x)$;
    choose $\text{host}(x) \in H_x \cap \{\text{host}(w), \text{first}(x)\}$;
    if *$\text{host}(x) \neq \text{host}(w)$* **then**
      add edge $\text{host}(w) \to \text{host}(x)$ to $\mathbf{v}$, adding nodes as necessary
    end
  end
end

Algorithm 2: Generating transmission trees.

Theorem 5. *Given a pathogen phylogeny Φ that is topologically consistent with the epidemiologic data, a transmission tree $\mathbf{v}$ is possible if and only if it can be generated using Algorithm 2.*

Proof. If **v** is a transmission tree simultaneously consistent with the epidemiologic data and Φ, then $\text{host}(x) \in H_x$ for each node x of Φ. Choose node $x \neq r_0$ and let $w = \text{parent}(x)$. By Lemma 3, $\text{host}(x) \in \{\text{host}(w), \text{first}(x)\}$. Therefore, $\text{host}(x) \in H_x \cap \{\text{host}(w), \text{first}(x)\}$. Since this is true for each such x, it is possible to generate **v** using Algorithm 2. Now suppose **v** a transmission tree generated by Algorithm 2. Choose a node $x \neq r_0$ in Φ and let $w = \text{parent}(x)$. There are two cases:

1. If $\text{host}(x) = \text{host}(w)$, there is no corresponding edge in **v**.
2. If $\text{host}(x) \neq \text{host}(w)$, then $\text{host}(x) = \text{first}(x)$ so $\text{host}(w)$ infected $\text{first}(x)$ by Assumption 5. Assume $\text{host}(w) \notin \mathcal{V}_{\text{host(x)}}$. Then $\text{host}(w) \in D_x$ by equation (1). Since $\text{host}(w) \in H_w \subseteq A_x$, we have $\text{host}(w) \in H_x$. But then $\text{host}(w)$ infected $\text{first}(x)$ by Lemma 2, which is a contradiction. Thus $\text{host}(w) \in \mathcal{V}_{\text{first}(x)}$, so the edge $\text{host}(w) \to \text{first}(x)$ is consistent with the epidemiologic data.

Since each edge in the **v** is consistent with the epidemiologic data, **v** is a possible transmission tree. □

```
Input: Rooted phylogeny Φ with known host(x) for each node x
Output: Branching time t_x for each node x
for node x in postorder traversal of Φ do
    if x is a leaf then set t_x to be the time pathogen x was sampled;
    else
        t_max = min_{y∈children(x)} t_y;
        choose t_x ∈ (t_host(x), t_max);
    end
end
```

Algorithm 3: Assigning branching times.

Theorem 6. *If a transmission tree is generated using Algorithm 2, then Algorithm 3 assigns a valid branching time to each internal node of the phylogeny. Any possible assignment of branching times can be generated this way.*

Proof. We must show that $t_{\text{host}}(x) < t_{\max}$ so t_x is chosen from a nonempty interval. For each child y of x, we have two posibilities:

1. If $\text{host}(y) = \text{host}(x)$, then $t_y > t_{\text{host}(x)}$ by construction.
2. If $\text{host}(y) \neq \text{host}(x)$, then $\text{host}(y) = \text{first}(y)$ by Lemma 3 so $\text{host}(x)$ infected $\text{first}(y)$ by Assumption 5. Thus, $t_{\text{host}(x)} < t_{\text{host}(y)} < t_y$.

Therefore, $t_{\text{host}(x)} < t_y$ for all y so $t_{\text{host}(x)} < t_{\max}$ and the algorithm will successfully find a branching time for each interior node x. Now suppose each interior node has been assigned a branching time t_x. If we traverse the phylogeny in postorder, we must have $t_x \in (t_{\text{host}(x)}, t_{\max})$ at each interior node x, so these times could be assigned using Algorithm 3. □
